# Application of Entropy Ensemble Filter in Neural Network Forecasts of Tropical Pacific Sea Surface Temperatures

**DOI:** 10.3390/e20030207

**Published:** 2018-03-20

**Authors:** Hossein Foroozand, Valentina Radić, Steven V. Weijs

**Affiliations:** 1 Department of Civil Engineering, University of British Columbia, Vancouver, BC V6T 1Z4, Canada; 2 Department of Earth, Ocean and Atmospheric Sciences, University of British Columbia, Vancouver, BC V6T 1Z4, Canada

**Keywords:** entropy ensemble filter, ensemble model simulation criterion, EEF method, bootstrap aggregating, bagging, bootstrap neural networks, El Niño, ENSO, neural network forecast, sea surface temperature, tropical Pacific

## Abstract

Recently, the Entropy Ensemble Filter (EEF) method was proposed to mitigate the computational cost of the Bootstrap AGGregatING (bagging) method. This method uses the most informative training data sets in the model ensemble rather than all ensemble members created by the conventional bagging. In this study, we evaluate, for the first time, the application of the EEF method in Neural Network (NN) modeling of El Nino-southern oscillation. Specifically, we forecast the first five principal components (PCs) of sea surface temperature monthly anomaly fields over tropical Pacific, at different lead times (from 3 to 15 months, with a three-month increment) for the period 1979–2017. We apply the EEF method in a multiple-linear regression (MLR) model and two NN models, one using Bayesian regularization and one Levenberg-Marquardt algorithm for training, and evaluate their performance and computational efficiency relative to the same models with conventional bagging. All models perform equally well at the lead time of 3 and 6 months, while at higher lead times, the MLR model’s skill deteriorates faster than the nonlinear models. The neural network models with both bagging methods produce equally successful forecasts with the same computational efficiency. It remains to be shown whether this finding is sensitive to the dataset size.

## 1. Introduction

Most data-mining algorithms require proper training procedures [1,2,3,4,5,6,7,8,9,10,11,12,13,14] to learn from data. The Bootstrap AGGregatING (bagging) method is a commonly used tool in the machine learning methods to increase predictive accuracy. Despite its common application, the bagging method is considered to be computationally expensive, particularly when used to create new training data sets out of large volumes of observations [15,16,17]. To improve the computational efficiency, Wan et al. [15] proposed a hybrid artificial neural network (HANN), while Kasiviswanathan et al. [17] combined the bagging method with the first order uncertainty analysis (FOUA). The combined method reduced the computational time of simulation for uncertainty analysis with limited statistical parameters such as mean and variance of the neural network weight vectors and biases. Wang et al. [16] showed that sub-bagging (SUBsample AGGregatING) gives similar accuracy but is computationally more efficient than bagging. This advantage is highlighted for Gaussian process regression (GPR) since its computational time increases in cubic order with the increase of data. Yu and Chen [18] compared different machine learning techniques and found that the fully Bayesian regularized artificial neural network (BANN) methods are much more time consuming than support vector machine (SVM) and maximum likelihood estimation (MLE)-based Gaussian process (GP) model. Table 1 provides a brief overview of studies that applied the bagging method in a range of different machine learning algorithms. 

The Entropy Ensemble Filter (EEF) method, as a modified bagging procedure, has been proposed recently by Foroozand and Weijs [19]. The EEF method uses the most informative training data sets in the ensemble rather than all ensemble members created by the conventional bagging method. The EEF method achieved a reduction of the computational time of simulation by around 50% on average for synthetic data simulation, showing a potential for its application in computationally demanding environmental prediction problems.

In this paper, the first application of the EEF method on real-world data simulation is presented. We test its application in forecasting the tropical Pacific sea surface temperatures (SST) anomalies based on the initially proposed neural-network model of Wu et al. [20]. We chose this particular application due to the numerous studies of the El Nino-southern oscillation (ENSO) phenomenon and its use for water resources management. The ENSO is the strongest climate fluctuation on time scales ranging from a few months to several years and is characterized by inter-annual variations of the tropical Pacific sea surface temperatures, with warm episodes called El Niño, and cold episodes, La Niña. As ENSO affects not only the tropical climate but also the extra-tropical climate [21,22], the successful prediction of ENSO is of great importance. One example is the Pacific Northwest of North America, where water management operations depend on the accuracy of seasonal ENSO forecasts. For the Columbia River hydropower system, the use of ENSO information, in combination with adapted operating policies, could lead to an increase of $153 million in expected annual revenue [23]. Successful long-term forecasts of ENSO indices themselves could increase forecast lead-times, potentially further increasing benefits from hydropower operations. Vu et al. [24] recently argued that information entropy suggests stronger nonlinear links between local hydro-meteorological variables and ENSO, which could further strengthen its predictive power. Also, recent drought in coastal British Columbia, Canada, has increased the need for reliable seasonal forecasts to aid water managers in, for example, anticipating drinking water supply issues.

Since the early 1980s, much effort has been allocated to forecasting the tropical Pacific SST anomalies with the use of dynamical, statistical and hybrid models [21,25,26]. Because of ENSO’s nonlinear features [20,21,27,28,29,30], many studies applied nonlinear statistical models such as a neural network (NN) model. Detailed comparisons between linear and nonlinear models in ENSO forecasts have been conducted in [2,20,30]. Wu et al. [20] developed a multi-layer perceptron (MLP) NN approach, where sea level pressure (SLP) field and SST anomalies over Tropical Pacific were used to predict the five leading SST principal components at lead times from 3 to 15 months. The performance of the MLP model, when compared to the multiple-linear regression (MLR) models, showed higher correlation skills and lower root mean square errors over most Nino domains. In this study, we incorporate the EEF method in both MLP and MLR models and evaluate their performance and computational efficiency relative to the original models. In addition to the original MLP model that uses Bayesian neural network (BNN), henceforth labeled as BNN model, we also test the MLP model that applies a cross-validation with Levenberg-Marquardt optimization algorithm [31,32,33], henceforth labeled as NN model. The main difference between BNN and NN models is their procedure to prevent overfitting. The NN model splits the provided data into training and validation and uses the early stop training procedure to prevent overfitting, while the BNN model uses all of the provided data points for training and uses weight penalty function (complexity penalization) to prevent overfitting (see [2,31,32,34] for details). 

This paper is structured as follows: in Section 2 we give a brief explanation of the EEF method and model structures, followed by a description of data, predictors, and predictands in Section 3. In Section 4 we present and discuss the results of the three models (MLR, BNN, NN) run with the conventional bagging method in comparison to the runs with the EEF method. Finally, a conclusion and outlook are presented in Section 5.

## 2. Methods

### 2.1. Entropy Ensemble Filter

The EEF method is a modified bagging procedure to improve efficiency in ensemble model simulation (see [19] for details). The main novelty and advantages of the EEF method are rooted in using the self-information of a random variable, defined by Shannon’s information theory [40,41] for selection of most informative ensemble models which are created by conventional bagging method [42]. Foroozand and Weijs [19] proposed that an ensemble of artificial neural network models or any other machine learning technique can use the most informative ensemble members for training purpose rather than all bootstrapped ensemble members. The results showed a significant reduction in computational time without negatively affecting the performance of simulation. Shannon information theory quantifies information content of a dataset based on calculating the smallest possible number of bits, on average, to convey outcomes of a random variable, e.g., per symbol in a message [40,43,44,45,46,47]. The Shannon entropy H, in units of bits (per symbol), of ensemble member M in a bagging dataset, is given by:

(1) $$H_{M}(X)=-\sum_{k=1}^{K}p_{x_{k}}\log_{2}p_{x_{k}},$$

where $p_{x_{k}}$ is the probability of occurrence outcome *k* of random variable *X* within ensemble member M. This equation calculates the Shannon entropy in the units of “bits” because logarithm’s base is 2. H gives the information content of each ensemble member in a discretized space, where the bootstrapped members are processed using K bins of equal bin-size arranged between the signal’s minimum and maximum values. These bin sizes are user-defined and chosen to strike a balance between having enough data points per bin and keeping enough detail in representing the data distribution of the time series. In this case, we chose K = 10. 

**Figure 1:**
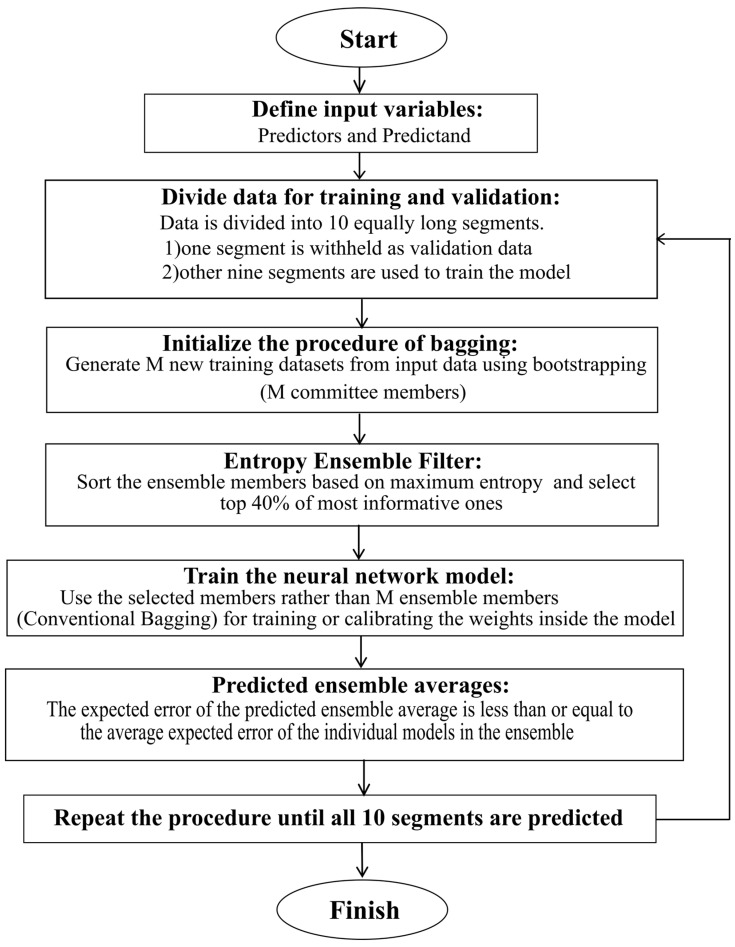
The flowchart of Entropy Ensemble Filter (EEF) method applied in the study.

Figure 1 illustrates the flowchart of the EEF procedure as applied in this study. The EEF method will assess and rank the ensemble members, initially generated by the bagging procedure, to filter and select the most informative ones for the training of the NN model. As it is expected in machine learning, the overall computational time depends roughly linearly on the number of retained ensemble members which potentially leads to significant time savings.

### 2.2. Models

Following Wu et al. [20], we adopt the same structure for the NN models used with both the conventional bagging and the EEF method. For details on the model’s training and cross-validation procedures to prevent overfitting, we refer to that paper [20]. The original model, a standard feed-forward multilayer perceptron neural network model with Bayesian regularization (BNN model) is run in MATLAB using the ‘trainbr.m’ function of the Neural Network Toolbox [34]. We introduce an additional NN model that applies the Levenberg-Marquardt optimization algorithm instead of Bayesian regularization and is run using the ‘trainlm.m’ MATLAB function (NN model) [32,48]. Finally, we run a multiple linear regression (MLR) model using the same 12 PCs as predictors. Following the recommendation of Wu et al. [20], in both neural network models, we optimize the number of hidden neurons (m) which is varying from 1 to 8 (the network architectures of 12-m-1) based on the correlation skill for each predictand out of five SST PCs. For all models we apply the following three schemes in order to produce the model ensemble runs: 

(1) MLR, NN, BNN scheme (following the original method in Wu et al [20]): Using the conventional bagging method, training data from the nine segments is randomly drawn multiple times in order to train the BNN and NN model separately. In each ‘bagging’ draw we include the model run into the ensemble only if the model’s correlation skill is higher than that of the MLR model, and its mean-square-error (MSE) less than that of the MLR model; if otherwise, the model run is rejected. This entire procedure is repeated until 30 ensemble members for each NN model are accepted. The ensemble average is then used as the final forecast result. With 10 segments for cross-validation and each segment producing 30 models, a total of 300 models are used for forecasting over the whole record.

(2) MLR_E_, NN_E_, BNN_E_ scheme: Using the EEF bagging method (Figure 1), training data from the nine segments is randomly drawn 12 times, producing 12 model ensemble for each data segment. We chose the model ensemble size to be 40% of the original one above, i.e., 12 out of 30, following the recommendations for EEF method application [19]. No selection criteria involving the comparison with the MLR model is applied here. A total of 120 models are used for forecasting over the whole record.

(3) MLR_rand_, NN_rand_, BNN_rand_ scheme: This scheme is the same as (2) except the conventional bagging scheme is used instead of the EEF method. This scheme mainly serves as a control run, i.e., for direct comparison of its performance with the scheme (2), both yielding the same total amount of 120 models over the whole period.

The above procedures are repeated until all five SST PCs at all lead times (3, 6, 9, 12 and 15 months) are predicted. For each lead time, we therefore have the ensemble-mean forecast from each of the nine models (MLR, MLR_E_, MLR_rand_, NN, NN_E_, NN_rand_, BNN, BNN_E_, BNN_rand_).

## 3. Data, Predictors, and Predictands

**Figure 2:**
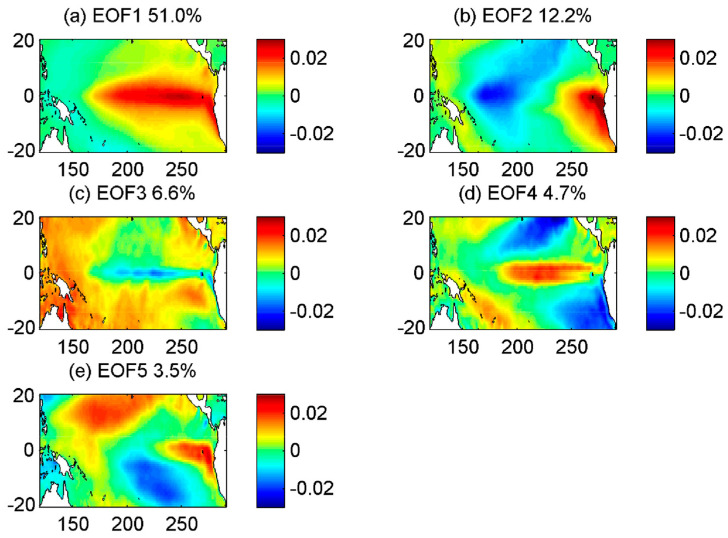
Spatial patterns (eigenvectors) of the first five PCA modes for the SST anomaly field. The percentage variance explained by each mode is given in the panel titles.

Monthly SST and SLP data in this study are from European Re-Analysis Interim (ERA-Interim) which is a global atmospheric reanalysis dataset [49] downloaded from the ECMWF website (https://www.ecmwf.int/en/forecasts/datasets/reanalysis-datasets/era-interim) for the tropical Pacific region (124° E–90° W, 20° N–20° S) at 0.75° × 0.75° for the period January 1979 to August 2017. Anomalies in both variables are calculated by subtracting their monthly climatology based on the 1979–2017 period. Following Wu et al. [20], we define the predictand as one of the five leading principal components (PCs) of the SST anomalies over the whole spatial domain, i.e., each of the five SST PCs is predicted separately. The corresponding spatial patterns of the eigenvectors (also called empirical orthogonal functions, EOFs) of the predictands, together explaining 80% of the total variance of the SST anomalies, are displayed in Figure 2. Note that our eigenvectors and SST PCs are somewhat different from those in Wu et al. [20] since their study used different reanalysis data and different time period (1948–2004). For the predictors, after applying a 3-month running mean to the gridded anomaly data of SLP and SST, a separate principal component analysis (PCA) is performed over the whole spatial domain with 7 SLP PCs and 9 SST PCs retained. These retained PCs are then separately normalized by dividing them by the standard deviation of their first PC. To set up the predictors’ structure, the 7 SLP PCs supplied at time leads of 0, 3, 6, and 9 months and the 9 SST PCs at time leads of 0 months are stacked together, altogether yielding 37 PC time series (4 × 7 SLP PCs and 9 SST PCs). Finally, another PCA is performed on the 37 PC time series to yield 12 final PCs that are used as the predictors in the models. As in Wu et al. [20], the lead time is defined as the time from the center of the period of the latest predictors to the center of the predicted period. The data record was partitioned into 10 equal segments (Figure 1), each 44 months long; one segment is withheld to provide independent data for testing the model forecasts, while the other nine segments are used to train the model. By repeating this procedure until all 10 segments are used for testing, we provide the forecast over the whole period. Following the recommendation in [20] for model evaluation criteria, we then calculate the correlation and root mean square error (RMSE) between the predicted SST anomalies and the corresponding target data (ERA-Interim) over the whole record. This is consistent with the Gaussianity assumptions underlying PCA and routinely employed in forecasting SST anomalies. Mean squared error (MSE) has been used inside of both neural network optimization procedures.

## 4. Results and Discussion

**Figure 3:**
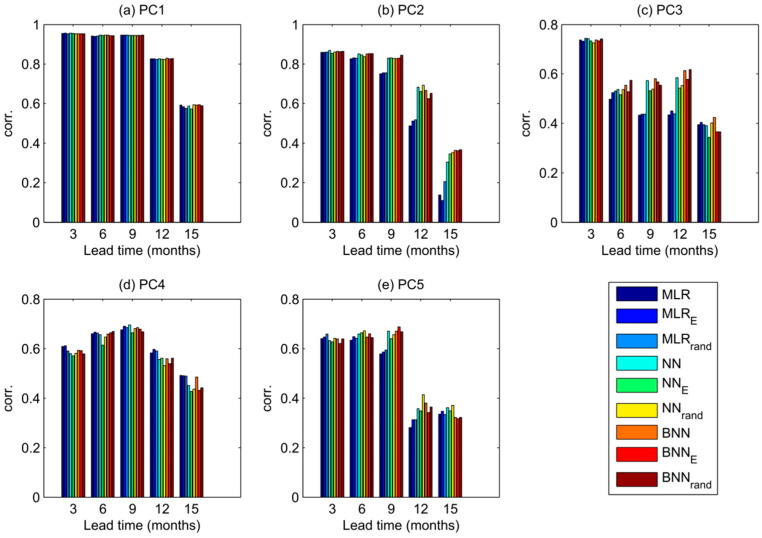
Correlation skill of predictions of the five leading principal components of the SST fields at lead times from 3 to 15 months for all 9 models.

We first inter-compare the performance of the nine modelling schemes across all five SST PCs and all five lead times by looking at the correlation between modelled and observed predictands (Figure 3). Note that, relative to Wu et al. [20] all our correlations are higher. It is important to note, though, that different time periods were used for the forecast, due to data availability. The pattern across PCs is the following: for the first PC, which carries most of the variance and spatially best resembles the ENSO pattern, all models perform equally well at all lead times with correlation coefficient greater than 0.9 for the lead times of 3–9 months. As we move to higher PCs in our experiments, with the exception of PC4, the neural network models out-perform the MLR model, especially at the lead times 12 and 15 months. The better performance of neural network models is particularly striking for the PC2 with lead times 12 and 15 months where the correlation of MLR model substantially drops from the value greater than 0.8 at the 3-month lead time to the value less than 0.2 at the 15-month lead time. This result indicates the importance of using nonlinear models for higher lead times, corroborating the findings in Wu et al. [20]. The BNN and NN models perform similarly well for PC1, PC2 and PC4, while the BNN model scores higher for PC3 and lower for PC5. The variation in skill between different methods seems to increase with increasing lead time and with higher PC modes. In general, the expected decrease of skill at higher lead times is visible throughout all PCs, except for PC4 where the predictability peaks at 9-months lead time. 

**Figure 4:**
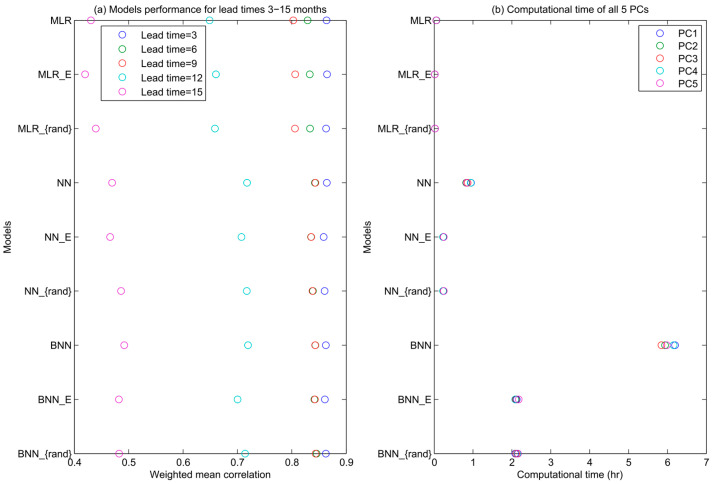
Weighted mean correlation and computational time for all models.

To assess the overall model performance, i.e., combining the skill across all five PCs, we derive the weighted mean correlation across all the PCs, for each lead time, assigning the weights to each PC mode according to the amount of variance explained by the mode (Figure 4a). The following patterns emerge: (1) all models perform equally well for the lead time 3 and 6 months; (2) MLR model’s skill drops at 9 months lead time more substantially than the skill of other models; (3) at the lead times of 9 and 15 months, the NN models outperform MLR models by roughly 0.05 difference in mean correlation (the difference between these models’ correlations is statistically significant, Steiger’s Z = 4.174 [50], *p* < 0.01 and Steiger’s Z = 1.73, *p* < 0.05 at the lead times of 9 and 15 months respectively); (4) the NN models all produce correlations very close to each other (within 0.01 difference in correlation); (5) at the 15-month lead time the BNN model is the best performing model; and (6) overall, EEF method achieved a significant reduction in computational time and performed well especially in the forecast at the first 3 lead times. However, its underperformance at 12 and 15 lead time can be regarded as a compromise on computational time-saving. 

Next, we look into the computational time for the models to produce the forecast for each PC mode (Figure 4b). The assessment is performed on the computer with 8 parallel quad-core processors (Intel^®^ Core™ i7-4790 CPU @ 3.60GHz × 8). As expected, the BNN simulation is the most computationally expensive with 30 hours runtime in total. Considering the similar performance among all the models at the lead time of 3 and 6 months, the faster algorithms (e.g., MLR model, NN model with EEF method or with random selection) have the computational advantage over the BNN model. It appears that the original Wu et al. [20] model, which relies on the selection criteria, i.e., the inclusion of ensemble members that perform better than their MLR equivalents, does not have an advantage over the modeling scheme without this selection criteria. Finally, the EEF method in BNN and NN models performs equally well and is equally computationally efficient as the application of random reduced ensemble selection, i.e., the conventional bagging. As expected, computation time is mainly driven by ensemble size, as training the models is the most computationally expensive step of the forecasting procedure. 

### Regional Forecast

**Figure 5:**
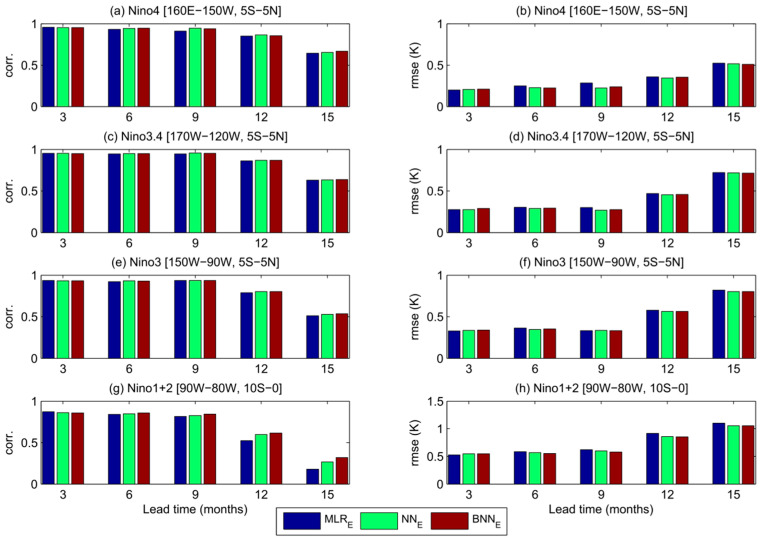
Correlation skills (**left column**) and RMSE scores (**right column**) of SST anomaly forecasts at lead times of 3–15 months for the Niño 4, Niño 3.4, Niño 3 and Niño 1+2 regions.

**Figure 6:**
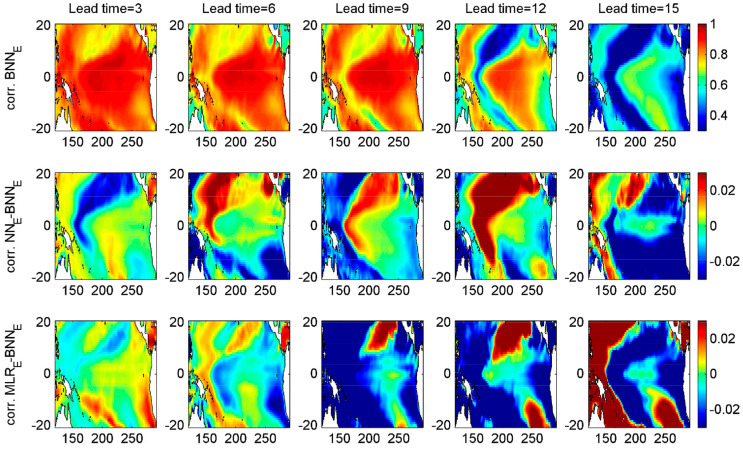
Forecast performance (correlation) per pixel of the forecast reconstructed from 5 leading principal components at lead times of 3–15 months for the period 1979–2017. Top row: BNN_E_ model, middle and bottom rows: improvement of performance of NN_E_ and MLR_E_ over BNN_E_.

To estimate the regional forecast skills over the whole tropical Pacific, SST anomaly fields were reconstructed from five predicted PCs multiplied by their corresponding EOF spatial patterns. We spatially averaged the reconstructed SST anomalies over the Niño 4 (160° E–150° W, 5° S–5° N), Niño 3.4 (170° W–120° W, 5° S–5° N), Niño 3 (150° W–90° W, 5° S–5° N), and Niño 1+2 (90° W–80° W, 10° S–0°) regions, and then computed the correlation skills and root mean squared error (Figure 5). We focus on the difference among the model performance with the EEF method (MLR_E_, NN_E_, and BNN_E_). Overall, their performances are at the same level at lead times of 3–12 months. The BNN model provides a better correlation skill than the other two models at 15 months lead time for all regions and especially for Niño 1+2 domain. We also look into the correlation skills spatially across each domain, i.e., correlation between reconstructed modeled and observed SST anomalies for each grid cell in the domain (Figure 6). At 15 months lead time, the large part of the domain is best simulated with the BNN model. There are significant variations between BNN and NN forecast skill at different lead times in central- northern equatorial region of the tropical Pacific. We also compared the time series of modeled vs observed SST anomalies averaged within the Niño 3 and Niño 1+2 regions at lead times of 3–15 months for the three models with the EEF method (see Appendix A). As expected, all models produced successful forecasts for the major El Niño and La Niña episodes at 3 months lead time. The BNN model outperformed other models at higher lead times, especially in Niño 1+2 region.

The spatial distribution of prediction skill was tested and compared for the various models at different lead times. Note that the performance of the BNN model relative to the NN model changes within the same region at different lead times (Figure 6). The same is true for the linear versus the nonlinear model. For example, for a lead time of 12 months, the nonlinear model outperforms the linear model in the Western Pacific, but not near Middle America, while the results for 15 month lead time are opposite. 

## 5. Conclusions

In this study, we performed sea-surface temperature (SST) forecasts over the tropical Pacific using both linear and nonlinear (neural network) models with different training and ensemble generation schemes. In addition to the conventional bagging scheme (randomly generated samples for model training), we applied the ensemble entropy filter (EEF) method. This method reduces the original model ensemble size, and thus computation time, while trying to maintain prediction quality by prioritizing the retention of the most informative training data sets in the ensemble. We incorporated the EEF method in a multiple-linear regression (MLR) model and two neural network models (NN and BNN) and evaluated their performance and computational efficiency relative to the same models when conventional bagging is used. The predictands were the principal components (PCs) of the first five modes of SST monthly anomaly fields over the tropical Pacific for the period 1979–2017. The models’ skills were tested for five different lead times: from 3 to 15 months, with 3 months increment. 

We show that all models perform equally well at the lead time of 3 and 6 months, while a significant drop in MLR model’s skill occurs at 9 month lead time and progressively deteriorates at 12 and 15 months lead time. At the higher lead times, the NN models outperform MLR models, while at the 15 months lead time the BNN model is the best performing model. Models with the EEF method perform equally well as the same models with the conventional bagging method with larger and equal ensemble sizes. Although the EEF method does not improve the correlation skill in the nonlinear forecast, it does not deteriorate it either. Considering that the EEF method selects only a portion of the data to be used in the forecasting, the improvement of computational efficiency with a minimum reduction in model skill makes this method attractive for the application on big datasets. For this particular case, however, the conventional bagging draws random ensembles that closely resemble the optimal ensembles from the EEF method. Thus, the neural network model with both bagging methods produced equally successful forecasts with the same computational efficiency. It remains to be shown, however, whether this finding is sensitive to the number of observations in the dataset. 

A limitation of this study is that we deal with deterministic predictions only. Using an ensemble of models in prediction would, in theory, lend itself to generate probabilistic forecasts if an appropriate post-processing scheme can be developed. This would then allow for information-theoretical analysis of prediction skill. Also, a more detailed analysis of variations in predictability could be undertaken, investigating links with slower modes of large scale circulation patterns, such as the Pacific Decadal Oscillation (PDO). This is best investigated in a practical prediction context. 

Future work will focus on further exploring the possibilities for forecasting streamflows in the Pacific Northwest, where seasonal forecasts can provide significant benefits for hydropower production, prediction of drinking water shortages and water management in general. Reducing computation time would enable smaller individual organizations to use seasonal forecasts tailored to their specific river basins and water resources management problems, rather than using agency issued ENSO index forecasts that may not always exploit the maximum information in the teleconnection to their most important variables. Also, the use of the ensemble prediction techniques to produce uncertainty estimates with the forecasts is a promising area of research that can inform risk-based decision making for water resources.

## Figures and Tables

**Table 1:** Examples of studies on machine learning algorithms with bagging methods and summary of their discussion on computational efficiency.

Authors (Year)	Machine Learning Method *	Computational Efficiency of the Bagging Method
Wan et al. (2016) [15]	HANN and BBNN	The bootstrapped NN training process is extremely time-consuming. The HANN approach is nearly 200 times faster than the BBNN approach with 10 hours runtime.
Liang et al. (2016) [35]	BNN and BMH	The bootstrap sample cannot be very large for the reason of computational efficiency.
Zhu et al. (2016) [27]	BNN	The proposed improvement in accuracy comes at the cost of time-consumption during the network training.
Gianola et al. (2014) [36]	GBLUP	Bagging is computationally intensive when one searches for an optimum value of BLUP-ridge regression because of the simultaneous bootstrapping.
Faridi et al. (2013) [37]	ANN	Each individual network is trained on a bootstrap re-sampling replication of the original training data.
Wang et al. (2011) [16]	ANN and GPR	Subagging gives similar accuracy but requires less computation than bagging. This advantage is especially remarkable for GPR since its computation increases in cubic order with the increase of data.
Mukherjee and Zhang (2008) [38]	BBNN	Dividing the batch duration into fewer intervals will reduce the computation effort in network training and batch optimisation. However, this may reduce the achievable control performance …
Yu and Chen (2005) [18]	BNN, SVM, and MLE-GP	Fully Bayesian methods are much more time consuming than SVM and MLE- GP.
Rowley et al. (1998) [39]	ANN	To improve the speed of the system different methods have been discussed, but this work is preliminary and is not intended to be an exhaustive exploration of methods to optimize the execution time.

* ANN (artificial neural network), BNN (Bayesian neural network), HANN (hybrid artificial neural network), BBNN (bootstrap-based neural network), BMH (bootstrap Metropolis-Hastings), GPR (Gaussian process regression), GBLUP (genomic best linear unbiased prediction), MLE-GP (maximum likelihood estimation-based Gaussian process), SVM (support vector machine) and V-SVM (virtual support vector machine).

**Figure A1:**
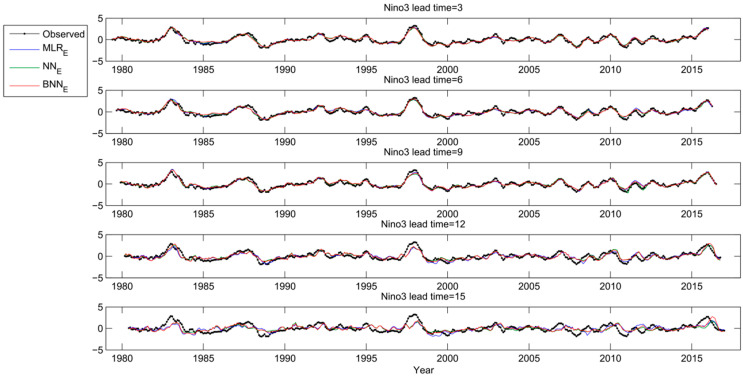
Time observed series of the Niño 3 SST anomalies as well as those predicted by different models at lead times of 3–15 months for the period 1979–2017.

**Figure A2:**
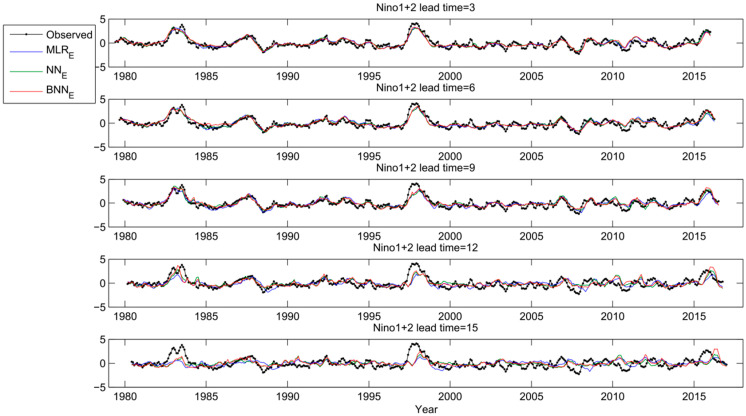
Time observed series of the Niño 1+2 SST anomalies as well as those predicted by different models at lead times of 3–15 months for the period 1979–2017.

## Appendix A. Results of Regional Forecast

In this appendix, the results are shown for predictions of various regional averages that are often used SST indices. The figures show forecasts for various lead times, reconstructed for the 1979–2017 period. Figure A1 shows the Niño 3 SST anomalies, while Figure A2 shows the Niño 1+2 SST anomalies.
